# Diagnostic Delay in HPV-Related Oropharyngeal Squamous Cell Carcinoma

**DOI:** 10.1055/s-0043-1767795

**Published:** 2024-02-05

**Authors:** Patrick O. McGarey, Osama Hamdi, Lane Donaldson, Kevin Zhan, Edwin F. Crandley, David D. Wilson, Austin J. Sim, Paul W. Read, Jonathan C. Garneau, Katherine L. Fedder, David C. Shonka, Mark J. Jameson

**Affiliations:** 1 Division of Head and Neck Oncologic and Microvascular Surgery, Department of Otolaryngology, Head and Neck Surgery, University of Virginia, Charlottesville, VA, United States; 2 Department of Radiation Oncology, University of Virginia, Charlottesville, VA, United States

**Keywords:** head and neck cancer, oropharyngeal squamous cell carcinoma, HPV, human papillomavirus viruses

## Abstract

**Introduction** Human papillomavirus-related (HPV + ) oropharyngeal squamous cell carcinoma (OPSCC) is increasing in incidence and presents diagnostic challenges given its unique clinical presentation.

**Objective** The purpose of the present study is to characterize the impact of the unique clinical presentation of HPV-related OPSCC on delays in diagnosis.

**Methods** Retrospective review of presenting symptoms and clinical characteristics of 284 patients with OPSCC treated from 2002–2014. Delay in diagnosis was defined as the presence of any of the following: multiple non-diagnostic fine needle aspirate (FNA) biopsies; two or more courses of antibiotic therapy; surgery with incorrect preoperative diagnosis; evaluation by an otolaryngologist without further workup; or surgery without definitive postoperative diagnosis.

**Results** p16+ tumors demonstrated a distinct clinical presentation that more commonly involved a neck mass (85.1% versus 57.3% of p16-; *p* < 0.001) and less frequently included odynophagia (24.6% versus 51.7% of p16-; *p* < 0.001). Patients who experienced diagnostic delay were more likely to have p16+ tumors (77.7% delayed versus 62.8% not delayed; *p* = 0.006). p16+ primary tumors were more likely to be undetectable by physical examination of the head and neck including flexible laryngoscopy (19.0% versus 6.7% of p16-; *p* = 0.007) and more frequently associated with nondiagnostic FNA biopsies of a cervical nodal mass (11.8% versus 3.4% of p16-, *p* = 0.03).

**Conclusions** Compared with non-HPV related OPSCC, the unique clinical presentation and characteristics of HPV+ OPSCC are associated with an increased incidence of diagnostic delay. Targeted education of appropriate care providers may improve time to diagnosis and treatment.

## Introduction

The Human Papillomavirus (HPV) was first identified in 1949, and molecular evidence for HPV as the etiologic agent of head and neck squamous cell carcinoma (HNSCC) appeared in 1983. 1 Since that time, the incidence of HPV-related (HPV + ) oropharyngeal squamous cell carcinoma (OPSCC) has increased dramatically, from roughly 16% of all OPSCCs in 1984 to 1988 to 72% by 2000 to 2004. 2 It has now become clear that HPV+ OPSCC has distinct characteristics from non-HPV related (HPV-) OPSCC.

Delay in the diagnosis of HNSCC is associated with poorer outcomes, 3 and is often stratified into patient delay (symptomatic onset to first health care professional appointment) and professional delay (first consultation to pathologic diagnosis). A subset of patients with HNSCC has a prolonged delay from onset of their symptoms to diagnosis; such delays can be attributed to a variety of social and disease factors. A large survey of HNSCC patients demonstrated that 9% of patients with squamous cell carcinoma (SCCA) outside of the oral cavity presented for an initial health care appointment 6 months or more after their initial symptoms. 4 A 2015 study from Canada cited several factors contributing to delays in diagnosis in HNSCC, including inappropriate treatment for infectious or reflux etiology, inappropriate reassurances by health care provider, lack of primary care physician knowledge of signs and symptoms of HNSCC, patient lack of knowledge of risk factors and symptoms of HNSCC, and patients taking alternative medicines. 5 A recent study cited that most patients with HPV- OPSCC visited at least two providers before diagnosis. Evaluation by three or more providers or being prescribed analgesia prior to diagnosis was associated with significant delays in diagnosis of nearly a year. 6 Additionally, patients with HPV+ OPSCC who were diagnosed at >12 months from symptom onset were more likely to have T4 disease and higher overall American Joint Committee on Cancer (AJCC) clinical stage at presentation than patients diagnosed < 12 months from symptom onset. 7

The aim of the present study was to further characterize HPV+ OPSCC by assessing for the presence of diagnostic delay compared with HPV- OPSCC and evaluating the role of various management factors that potentially impact time to diagnosis.

## Materials and Methods

The present study has undergone formal review and approval by our institutional review board (IRB), with the approval number 13457. We reviewed 554 cases of OPSCC treated from January 2002 through December 2014. p16 immunohistochemistry (IHC) was used as an indicator of HPV status for all patients from biopsy or surgical tissue when available. Since 2008, p16 staining has been routinely performed in HNSCC biopsies or surgical specimens; a tissue micro array (TMA) was used to obtain p16 status for a portion of patients treated prior to this date. A total of 213 cases were excluded due to lack of p16 IHC. A total of 57 cases were excluded due to inadequate documentation or history of prior HNSCC. Ultimately, 284 cases were included. All patients underwent treatment planning by the Head and Neck Tumor Board. Medical records were reviewed to obtain patient demographics, smoking and alcohol history, presenting symptoms, medical comorbidities, and to assess for delays in diagnosis. Univariate chi-squared, *t* -test, and Fisher exact tests were utilized to compare these characteristics for p16+ versus p16- OPSCC. All tumors were staged according to AJCC 7th Edition staging guidelines. Alcohol use was determined by consumption of > 5 alcoholic beverages per week. 6 To assess factors independently associated with diagnostic delay in this population, a multivariable logistic regression analysis was performed (IBM SPSS Statistics for Windows, IBM Corp., Armonk, NY, USA) using characteristics that were statistically more likely to be present in patients who experienced diagnostic delay versus those who did not.

**Fig. 1 Flowchart for the diagnosis of patients with OPSCC.**
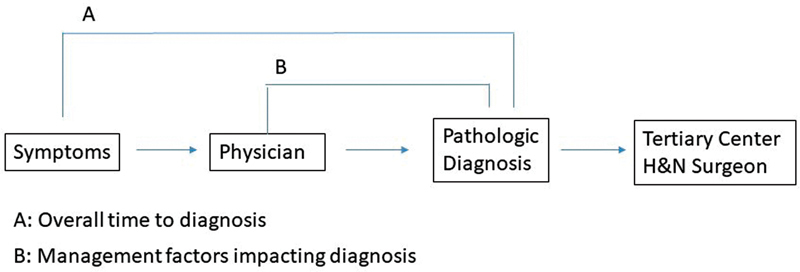
The time between patients beginning to experience symptoms and the arrival at a pathologic diagnosis was termed overall time to diagnosis. The time between presentation to a physician and the arrival at a pathologic diagnosis highlights the management factors impacting diagnosis.

Diagnostic delay was assessed in two ways. First, overall time to diagnosis ( Fig. 1A ) was measured in months from the time the patient began experiencing symptoms until a pathologic diagnosis was made. Overall time to diagnosis was used as a continuous variable with higher values representing greater diagnostic delay. Second, management factors impacting diagnosis ( Fig. 1B ) were evaluated as a categorical variable, with any of the following representing diagnostic delay: 1) multiple nondiagnostic fine needle aspirate (FNA) biopsies; 2) two or more courses of antibiotic therapy; 3) surgery with incorrect preoperative diagnosis; 4) evaluation by an otolaryngologist without further workup; or 5) surgery without definitive postoperative diagnosis.

## Results

### Patient and Tumor Characteristics

Table 1 shows patient characteristics for p16+ and p16- cohorts. There was no significant difference in the mean age at diagnosis or gender distribution between p16+ (58.6 years, old 22.5% female) and p16- OPSCC (58.2 years old, 16.4% female). A total of 93.3% of patients with p16+ tumors were white, compared with only 66.3% of the p16- group ( *p* < 0.001). In patients with p16+ tumors, there were significantly lower rates of tobacco use (65.1 versus 92.1% of p16-; *p* < 0.001) and alcohol use defined as > 5 drinks weekly (30.3 versus 61.8% of p16-; *p* < 0.001).

**Table 1:** Patient characteristics

	**All**		***p-value***	**p16 Status**				***p-value***
				**p16-**		**p16**		
**Patients**	284	(100.0%)		89	(31.3%)	195	(68.7%)	
**Age**								
Mean Age (years old)	**58.3**	***n *** **= 284**	0.21 ^a^	58.6		58.2		0.69 ^a^
Mean Age with Dx Delay (years old)	**57.0**	***n *** **= 112**		57.6		56.8		0.70 ^a^
**Gender**								
Females	52	(18.3%)	−	20	(22.5%)	32	(16.4%)	0.22 ^b^
Males	232	(81.7%)		69	(77.5%)	163	(83.6%)	
**Tobacco**								
Tobacco user	209	(73.6%)	−	82	(92.1%)	127	(65.1%)	** < 0.001 ^b^**
Tobacco nonuser	75	(26.4%)		7	(7.9%)	68	(34.9%)	
**Alcohol**								
Use	114	(40.1%)	−	55	(61.8%)	59	(30.3%)	** < 0.001 ^b^**
Nonuse	170	(59.9%)		34	(38.2%)	136	(69.7%)	

(a) Student *t* -test.

(b) Chi-squared test.

As shown in Table 2 , 99.5% of p16+ tumors originated in the base of the tongue (33.3%) and tonsils (66.2%); 13 of 14 OPSCC cases originating outside of these locations were p16-. p16+ tumors were more likely to present with smaller (T1-T2) primaries (72.3 versus 59.6% of p16-; *p* = 0.03) and with more advanced nodal stage (90.8% N+ versus 74.2% of p16-).

**Table 2:** Tumor characteristics

	All		p16 Status				p *-value*
			p16-		p16		
	284	(100.0%)	89	(31.3%)	195	(68.7%)	
**Primary Site**							
Base of tongue	88	(31.0%)	23	(25.8%)	65	(33.3%)	** < 0.001 ^a^**
Tonsil	182	(64.1%)	53	(59.6%)	129	(66.2%)	
Other ^b^	14	(4.9%)	13	(14.6%)	1	(0.5%)	
**Primary Stage**							
T1/2	194	(68.3%)	53	(59.6%)	141	(72.3%)	** 0.03 ^a^**
T3/4	90	(31.7%)	36	(40.4%)	54	(27.7%)	
**Nodal Stage**							
N0	41	(14.4%)	23	(25.8%)	18	(9.2%)	** < 0.001 ^a^**
N1/2a	62	(21.8%)	12	(13.5%)	50	(25.6%)	
N2b/2c/3	181	(63.7%)	54	(60.7%)	127	(65.1%)	
**Stage Group**							
I/II	23	(8.1%)	13	(14.6%)	10	(5.1%)	** 0.007 ^a^**
III/IV	261	(91.9%)	76	(85.4%)	185	(94.9%)	

(a) Chi-squared test.

(b) Soft palate, lateral oropharyngeal wall, posterior oropharyngeal wall.

### Presenting Symptoms

Table 3 summarizes the presenting symptoms for patients with p16- and p16+ OPSCC. Compared with patients with p16- tumors, patients with p16+ OPSCC presented more often with neck mass (85.1 versus 57.3%; *p* < 0.001) and less often with throat pain/odynophagia (24.6 versus 51.7%; *p* < 0.001) or weight loss (19.0 versus 41.6%; *p* < 0.001). Although they did not achieve statistical significance, otalgia (33.3 versus 44.9%; *p* = 0.06) and shortness of breath (4.1 versus 10.1%; *p* = 0.06) were also noted less frequently in patients with p16+ tumors.

**Table 3:** Presenting symptoms and diagnostic process

	p16-		p16		*p-value*
	*n* = 89		*n* = 195		
**Neck Mass**	50	(56.2%)	166	(85.1%)	** < 0.001 ^a^**
**Throat Pain**	45	(50.6%)	48	(24.6%)	** < 0.001 ^a^**
**Dysphagia**	33	(37.1%)	64	(32.8%)	0.48 ^a^
**Otalgia**	40	(44.9%)	65	(33.3%)	0.06 ^a^
**Weight loss**	37	(41.6%)	37	(19.0%)	** < 0.001 ^a^**
**Dyspnea**	9	(10.1%)	8	(4.1%)	0.06 ^b^
**Fatigue**	8	(9.0%)	12	(6.2%)	0.45 ^b^
**Voice Change**	15	(16.9%)	28	(14.4%)	0.59 ^a^
**Dysguesia**	1	(1.1%)	4	(2.1%)	1.0 ^b^
**Overall Time to Diagnosis**					
Mean symptom onset until pathology diagnosis in months (SD)	4.75	(5.03) ^c^	4.65	(5.96) ^d^	0.90 ^e^
**Management Factors Impacting Diagnosis**					
None	64	(71.9%)	108	(55.4%)	** 0.008 ^a^**
Any	25	(28.1%)	87	(44.6%)	
**Occult Primary**	6	(6.7%)	37	(19.0%)	** 0.007 ^b^**
**Cystic Lymph Node(s)**	1	(1.5%) ^f^	17	(9.6%) ^g^	** 0.02 ^b^**
**Necrotic Lymph Node(s)**	40	(60.6%) ^f^	22	(12.4%) ^g^	** 0.05 ^a^**
**Nondiagnostic FNA (any)**	3	(3.4%)	23	(11.8%)	** 0.02 ^b^**
**Nondiagnostic FNA (number)**					
0	86	(96.6%)	172	(88.2%)	0.74 ^a^
1	2	(2.2%)	18	(9.2%)	
≥2	1	(1.1%)	5	(2.6%)	

Abbreviations: FNA, fine needle aspirate; SD, standard deviation.

(a) Chi-squared test.

(b) Fisher exact test.

(c) Reported for 71 patients.

(d) Reported for 132 patients.

(e) Student *t* -test.

(f) Of 66 N+ patients.

(g) Of 177 N+ patients.

### Diagnostic Findings

A summary of the diagnostic process is presented in Table 3 . Patients with p16+ OPSCC experienced diagnostic delay more frequently due to management factors prior to presentation to the Head and Neck Oncologic Surgery service (44.6 versus 28.1% of p16-; *p* = 0.008) ( Fig. 1B ). p16+ primary tumors were more likely to be undetectable by physical examination of the head and neck including flexible laryngoscopy (19.0 versus 6.7% of p16-; *p* = 0.007). Patients with p16+ OPSCC more often had cystic lymph node metastasis (8.8 versus 1.1% of p16-; *p* = 0.02) while patients with p16- tumors had a higher rate of necrotic nodes (44.9 versus 33.3% of p16 + ; *p* = 0.05). Patients with p16+ OPSCC underwent more frequently nondiagnostic FNA biopsy of a cervical nodal mass (11.8 versus 3.4% of p16-; *p* = 0.03). Five patients in the p16+ group underwent ≥ 2 nondiagnostic FNA biopsies.

Table 4 compares patients who did versus who did not experience diagnostic delay due to management factors. There was no significant difference in age, gender, or race. Patients who experienced diagnostic delay were 1) more likely to have p16+ tumors (77.7% delayed versus 62.8% not delayed; *p* = 0.006); 2) less likely to consume alcohol (33.0% delayed versus 44.8% not delayed; *p* = 0.047); 3) more likely to exhibit weight loss (33.0% delayed versus 21.5% not delayed; *p* < 0.001); 4) more likely to have an occult primary tumor (25.0% of delayed versus 8.7% of not delayed; *p* < 0.001); and 5) more likely to undergo a nondiagnostic FNA (17.0% delayed versus 4.1% not delayed; *p* = 0.001). Using the significant associations shown in Table 3 , a multivariate analysis was performed to detect independently associated variables and the results are shown in Table 5 . Alcohol consumption was not interpedently associated with diagnostic delay. Weight loss, occult primary tumor, any nondiagnostic FNA, and p16 positivity were independently associated with delay in diagnosis ( Table 5 ).

**Table 4:** Analysis of factors potentially associated with diagnostic delay

	No diagnostic delay		Diagnostic Delay		*p-value*
	*n* = 173		*n* = 112		
**Neck Mass**	130	(75.6%)	86	(76.8%)	0.817
**Throat Pain**	57	(33.1%)	36	(32.1%)	0.86
**Dysphagia**	55	(32.0%)	42	(37.5%)	0.34 ^a^
**Otalgia**	64	(37.2%)	41	(36.6%)	0.92 ^a^
**Weight loss**	37	(21.5%)	37	(33.0%)	** < 0.001 ^a^**
**Dyspnea**	11	(6.4%)	6	(5.4%)	0.72 ^b^
**Fatigue**	11	(6.4%)	9	(8.0%)	0.61 ^b^
**Voice Change**	26	(15.1%)	28	(15.2%)	0.99 ^a^
**Dysguesia**	2	(1.2%)	3	(2.7%)	0.38 ^b^
**P16 status**					
P16-	64	(37.2%)	25	(22.3%)	** 0.006 ^a^**
P16+	108	(62.8%)	87	(77.7%)	
**Occult Primary**	15	(8.7%)	28	(25.0%)	** < 0.001 ^b^**
**Cystic Lymph Node(s)**	7	(4.1%)	17	(9.8%)	0.074 ^b^
**Necrotic Lymph Node(s)**	67	(39.0%)	38	(33.9%)	0.39 ^a^
**Nondiagnostic FNA (any)**	7	(4.1%)	19	(17.0%)	** 0.001 ^b^**
**Tobacco Use**	132	(77.2%)	77	(68.8%)	0.12
**Alcohol Use**	77	(44.8%)	37	(33.0%)	**0.047**

Abbreivation: FNA, fine needle aspirate.

(a) Chi-squared test.

(b) Fisher exact test.

(c) Student *t* -test.

**Table 5:** Multivariate analysis of factors associated with diagnostic delay ^a^

	OR	95%CI	*p-value*
p16+	2.08	1.13–3.94	0.022
Weight Loss	2.80	1.55–5.16	0.001
Occult Primary Tumor	2.88	1.40–6.07	0.005
Nondiagnostic FNA	3.60	1.41–10.08	0.010
Alcohol consumption	0.82	0.47–1.44	0.495

Abbreviations: CI, confidence interval; FNA, fine needle aspirate; OR, odds ratio.

## Discussion

Our findings corroborate previous studies demonstrating that HPV+ OPSCC occurs more often in patients who are white, nonsmokers, and nonalcohol users. In addition, p16+ OPSCC presented more often with a neck mass and less often with odynophagia and weight loss compared with p16- OPSCC. We found no significant difference in rates of dysphagia. Our results also concur with previous studies demonstrating the increased incidence of cystic lymph node metastasis in HPV+ OPSCC. Weight loss, occult primary tumor, nondiagnostic FNA, and p16 positivity were independently associated with diagnostic delay.

Our study examines a large cohort of OPSCC patients and helps characterizing the differences in presenting symptoms and evaluation in HPV+ OPSCC. In addition, we identified several clinical events that delay diagnosis in the HPV+ OPSCC population, including nondiagnostic FNA, repeated trials of empiric therapy, and, occasionally, open surgical procedures prior to defining the diagnosis. Studies of delays in diagnosis encounter many limitations. Calculating time from presenting symptoms to initial consultation is difficult and subject to recall bias. We elected to use both temporal and clinical markers to evaluate diagnostic delays in our patients. While deviating from a purely time-based measure may be less objective, this allows a focus on specific clinical events that contribute to delays in time to diagnosis. Despite this approach, the study is retrospective and limited by the quality and completeness of prior documentation, and is potentially impacted by multiple uncontrolled variables, including clinician charting, introduction of new treating and/or referring clinicians during the study period etc. Regardless of the limitations, the study demonstrates that patients with HPV+ OPSCC are at risk for delay in diagnosis and treatment. It also suggests that targeted education of appropriate care providers around the unique presentation of HPV+ OPSCC as compared with the “classic” HPV- OPSCC could improve time to diagnosis and treatment.

Dhooge et al. reviewed 127 cases of HNSCC from all subsites but with roughly 50% laryngeal SCCa and found that 15.7% of the patients had a time interval from symptom to diagnosis > 12 months. Subsite location can affect time to diagnosis; several studies have found that laryngeal SCCa is associated with a longer delay in diagnosis compared with oropharyngeal cancer. 9 10

Crucial in the interpretation of these results in the context of other studies is the distinction between statistical and clinical significance. Kowalski et al. studied the time interval needed for clinical upstaging in 69 cases who experienced delays in treatment compared with 138 controls who were treated shortly after diagnosis in Brazil. The delay in treatment of the cases was due to initial treatment refusal (53.7%) or a long waiting period for hospital admission (40%). The shortest median time for clinical upstaging was for resectable stage IV to unresectable stage IV, which took an average of 3.6 months. The median time for clinical upstaging from stage I to stage II was 6.3 months; and from III to IV, it was 8.5 months. 11 This data provides a gross measure for the degree of delay in diagnosis which is clearly clinically significant.

Goy et al performed a systematic review of studies evaluating diagnostic delay in head and neck cancer. Given the heterogeneity of cancer site and measurement of delay among the 27 studies reviewed, the group did not aggregate the data as a meta-analysis. There were five studies evaluating pharyngeal cancer. Three studies demonstrated no relationship between diagnostic delay and more advanced stage at diagnosis, two studies found a positive relationship between delay in diagnosis and more advanced stage at diagnosis. Although very few studies (when including all cancer sites) demonstrated that delay in diagnosis was associated with more advanced stage at diagnosis, delay in diagnosis did predict survival in many of the studies examined. This suggests that the TMN staging system is perhaps not sensitive enough to detect the actual clinical effects of delays in diagnosis. 3

Seoane et al. performed a systematic review and meta-analysis of the impact of diagnostic delay on survival in HNSCC, which included 10 studies. The pooled data showed equivocal results, with a trend between patient/professional delay and worsened mortality which did not reach statistical significance. For all subsites, total delay was not associated with worsened mortality (pooled relative risk [RR]: 1.04; confidence interval [CI]: 1.01–1.07), but pharyngeal site had the highest association between diagnostic delay and mortality (pooled RR 1.68; CI: 1.22–2.31). 12 Despite these equivocal results, it is commonly agreed in the field that more rapid diagnosis of malignancy will reduce treatment burden and morbidity and improve survival. More recently, Schutte et al. performed a systematic review on the impact of time to diagnosis and treatment in head and neck cancer, which included 51 studies. They found delay in HNC diagnosis and treatment is associated with higher stage and worse survival. They found that an increased delay was associated with decreased overall survival in 8 of 14 studies investigating the effect of time delays on survival. Similar outcomes were found for disease-specific survival. 13

Huang et al. evaluated factors that contribute to delay in diagnosis in HPV- and HPV+ OPSCC. In alignment with our findings, their review of 304 patients with OPSCC showed HPV+ OPSCC presented more often with asymptomatic neck mass and underwent more nondiagnostic FNAs. Nearly 10% of patients with HPV+ OPSCC in their cohort required > 12 months from first presentation to diagnosis. They attributed the delay in ⅓ of this subset to be clinician-related and ⅔ to be patient related. 14 Similarly, Davis et al. reported 6 patients with delayed diagnosis of HPV+ OPSCC, where nondiagnostic or negative FNAs ( *n* = 2) and negative direct laryngoscopy and biopsy ( *n* = 1) were taken as sufficient evidence of a benign cyst and further biopsies were deferred. They reported a median time from development of a unilateral neck mass to presentation for alternate opinion and suspected HPV-OPSCC as 42 months, ranging from 3 months to 7 years. 15

HPV+ OPSCC is known to have distinct tumorigenesis from HPV- OPSCC. 16 HPV+ OPSCC is characterized by a younger patient population and association with nonsmoking status, 17 18 a propensity to cause malignancy in oropharyngeal sites compared with other sites of the head and neck, 19 20 smaller primary tumor size, 21 cystic lymph node metastasis, 22 improved response to radiotherapy, 23 24 improved survival compared with HPV- OPSCC, 17 25 and decreased risk of second primary malignancy. 26 27

Delays in diagnosis in HPV-related OPSCC is likely multifactorial. First, the presenting demographics, tumor characteristics, and symptoms of HPV+ OPSCC are distinct from “classic” HPV- disease. HPV+ OPSCC presents more often with a neck mass and less often with odynophagia, otalgia, dyspnea, or weight loss. Many patients with HPV+ tumors are nonsmokers. When otherwise healthy patients present with a neck mass and without throat pain, they are more likely to be treated for an infectious process, than immediately biopsied or referred to a specialist. If the neck mass persists after antibiotic therapy, imaging may show a cystic node, which can be mistaken for a branchial cleft cyst or other developmental anomaly, despite the fact that these generally present earlier in life. The overall incidence of malignancy in a cystic neck mass is only between 10 and 22%, 28 29 but increases to as high as 80% in patients > 40 years old. 30 Therefore, according to their 2017 clinical practice guidelines on the evaluation of a neck mass in adults, the American Academy of Otolaryngology – Head and Neck Surgery recommends work-up of a cystic neck mass until a definitive diagnosis is obtained. 31

As shown by multivariable analysis, both the presence of nondiagnostic FNA and an occult primary tumor were independently associated with diagnostic delay. p16+ OPSCC was more often associated with one or more nondiagnostic FNA lymph nodes biopsies, which could result in further delay in referral or give false reassurance. HPV+ OPSCC primary tumors were also more frequently undetectable on physical exam and flexible laryngoscopy at the time of evaluation by an Otolaryngologist, potentially resulting in further delay despite specialist evaluation. Franco et al. performed a retrospective review of 100 sequential patients with HNSCC and found the most common reason for diagnostic delay was a prolonged wait to evaluation in being seen in the otolaryngology clinic after referral placement (28.6%) followed by diagnostic error by the referring physician (22%) and delayed referral of a symptomatic patient to the otolaryngology clinic (16.2%); these findings further highlight the importance of raising awareness and targeted education of appropriate care providers. 32

## Conclusions

Patients with HPV+ OPSCC experienced a significantly higher rate of diagnostic delay that is likely associated with inappropriate treatment (such as antibiotics) and procedures (repeat FNA, excisional biopsy) and delays in time to treatment. Additional research and targeted education of appropriate care providers may improve time to diagnosis in this population.
